# Tracing evolution of spatio-temporal dynamics of the cerebral cortex: cortico-cortical communication dynamics

**DOI:** 10.3389/fnsys.2014.00076

**Published:** 2014-05-05

**Authors:** Per E. Roland, Claus C. Hilgetag, Gustavo Deco

**Affiliations:** ^1^Department of Neuroscience and Pharmacology, Faculty of Health Sciences, University of CopenhagenCopenhagen, Denmark; ^2^Department of Computational Neuroscience, University Medical Center Hamburg-EppendorfHamburg, Germany; ^3^Department of Health Sciences, Boston UniversityBoston, MA, USA; ^4^Computational Neuroscience Group, Department of Technology, University of Pompeu FabraBarcelona, Spain

**Keywords:** action potential transmission, connectivity models, multi-area voltage sensitive dye recordings, EEG, MEG

A considerable number of axons from neurons in one cortical area end up on other cortical areas. When one neuron in one cortical area sends an action potential to target neurons in other cortical areas, this is a realization of a cortico-cortical communication. Sensory perception, thinking, and planning of a specific behavior, all rely on the evolution of cortico-cortical communications. The action potentials change the membrane potentials in the target neurons and, in turn, may excite these neurons to produce action potentials and complex patterns of excitation and inhibition in *their* targets. We launched the *special research topic of cortico-cortical communication dynamics* to invite contributions that would cast light on such evolution of spatio-temporal action potential and membrane potential dynamics in the cerebral cortex.

The contributions were theoretical models, human EEG, and MEG data and data-driven models, and *in vivo* experimental data from animals accounting for specific aspects of cortico-cortical communication dynamics.

In a recent *in vitro* experiment, Branco et al. (2010) show that single dendrites of pyramidal layer 2–3 neurons depolarize more and have larger Ca^2+^ influx when their depolarization progresses toward the soma, than when depolarization progresses away from the soma. Kiebel and Friston (2011) construct a (developmental) model of the pruning of single synapses and show that they can reproduce the findings of Branco et al. (2010) if the self-organizing pruning follows a Bayesian and information theory derived principle of minimization of free energy. Cortico-cortical communication dynamics can only be comprehensively studied *in vivo. In vivo*, the neurons and their dendrites are in a high conductance state (Destexhe et al., 2003), and the propagation of depolarizations to the soma and action potential generation may thus be difficult to predict (Williams and Mitchell, 2008). This does not exclude, however, that the model of Kiebel and Friston (2011) may be appropriate in early development and in the formation of cortio-cortical synapses. The pruning of synapses under development and hence the formation of the adult cortical network is the theme of the contribution of van den Bergh et al. (2012). Their model departs from a random network. This network is subsequently shaped by spontaneous ongoing spike activity. After a while the random structure disappears and many small-world sub-networks emerge. As van den Bergh et al. (2012) show, this only happens if the connectivity in the network is larger than a critical value. This is interesting as the developing brain has many cortico-cortical connections that disappear at later stages.

As pointed out in a critical review of cortico-cortical communication dynamics, there are many obstacles precluding the tracing the ms by ms evolution of the spatio-temporal dynamics of the cortex (Roland et al., 2014). Therefore examination of the spatio-temporal dynamics in biologically plausible computational models of neurons may be one way to develop experimentally testable hypotheses. Li and Zhou (2011) made a computational model of neurons in two inter-connected cortical areas. The duration of the delays in communication and the distribution of inhibition in the local network determined whether the neurons would spike in phase or in anti-phase and whether interactions between slow and fast membrane oscillations would produce anti-phase spiking. These findings are pertinent for the hypothesis on cortico-cortical communication through coherence (Fries, 2009).

Facing the obstacles of tracing the spatio-temporal dynamics of cortico-cortical communications at the cellular scale, many scientists choose to study membrane electrical activity at the scale of large neuron populations, and from EEG and MEG signals try to infer putative routes of communication. Banerjee et al. (2012) discuss these methods and point out that there is no consensus as to what constitutes a large-scale network. Further, they show how MEG measurements may be interpreted by combining the empirical analysis with large-scale models of biologically realistic membrane activity. This is what is done in the contributions by Misic et al. (2011) and Vakorin et al. (2011). Their results show that time delays and the number of connections between sources, of MEG signals or EEG signals, contribute to the relation between variance in the signals and information transfer between the sources (Misic et al., 2011; Vakorin et al., 2011).

At the mesoscopic scale one can observe changes in the membrane potentials with voltage sensitive dyes, local field potentials and combine this with recordings of action potentials from a few neurons or single neurons in experimental animals. Harvey and Roland (2013) demonstrate both forward spatiotemporal population membrane dynamics in higher visual areas that after 50 ms was followed by backward propagation of net-excitation from these areas in experiments with objects moving in the visual field. Vinnik et al. (2012) examined the communications from the auditory cortex to the hippocampus and show that the access to fire hippocampal neurons is state dependent. Sleep favors fast reactions of the hippocampal neurons to the extent as only seen for novel sounds in awake animals (Vinnik et al., 2012). Civillico and Contreras (2012) examined how the communication from the thalamus to the barrel cortex is affected by the state of the neurons in the barrel cortex. When the cortical neurons were in an up-state, the local field potentials, the membrane potential increases, and the multiunit activity evoked by a whisker stimulus was smaller than when the whisker stimulus was given just during the early transition from a down-state to an up-state (Civillico and Contreras, 2012).

If one wants to understand how the cerebral cortex works one must be able to trace the evolution of the spatio-temporal transmission of action potentials and membrane conductances down to the cellular scale. As the critical review concludes, this is not possible yet. Assume that a full connectome of the mouse cerebral cortex exists (Bohland et al., 2009). This might help in finding the target neurons in other areas for a given neuron. However, it still remains to identify that source neuron spiking in an experiment and measure the membrane potential changes induced by that neuron on each of the target neurons, as each target neuron may have 1000 other source neurons. One may argue that if this multidimensional cellular dynamics should have any impact on perception and behavior, the dynamics of action potentials and membrane potential dynamics at more coarse scales should organize to make such impacts. The contributions to this special issue are fine examples of the many contemporary attempts to advance theoretical knowledge of cortico-cortical communication dynamics, provide testable hypotheses in this field, and test these hypotheses at the microscopic, mesoscopic, and macroscopic scales.

## Conflict of interest statement

The authors declare that the research was conducted in the absence of any commercial or financial relationships that could be construed as a potential conflict of interest.
